# A Novel Management Platform Based on Personalized Home Care Pathways for Medicine Management and Rehabilitation of Persons With Parkinson's Disease—Requirements and Implementation Plan of the Care-PD Program

**DOI:** 10.3389/fneur.2021.672208

**Published:** 2021-05-25

**Authors:** Song Gao, Yuning Hou, Renyan Ma, Keneilwe Kenny Kaudimba, Lingjing Jin, Han Wang, Ru Wang

**Affiliations:** ^1^School of Kinesiology, Shanghai University of Sport, Shanghai, China; ^2^Department of Neurology, Tongji Hospital, Tongji University, Shanghai, China; ^3^Department of Neurology, Peking Union Medical College Hospital, Beijing, China

**Keywords:** Parkinson's disease, care-PD program, telemedicine, home care plan, medicine management, rehabilitation

## Abstract

As the percentage of the aging population increases, the incidence of Parkinson's disease (PD) in China is increasing year by year. PD is both a public health and social problem facing the government and society as a whole. Persons with PD need reasonable medication management and rehabilitation strategies after a clear diagnosis. A proper home care plan can effectively slow the progression of PD. However, people with PD lack an effective way to manage their illnesses and cannot achieve the recommended clinical path in a family environment. Medication management, condition monitoring, and rehabilitation training are important components of the home care plan for PD. Persons with PD require strategies that delay the development of the disease and to adhere to treatment, which would contribute to improving their quality of life. Thus, we developed a small program called Care-PD to build a medicine management and service platform for PD. The development of Care-PD is a multi-dimensional model designed for PD, which is funded by the National Key R&D Program of China (No. 2018YFC1314700), and includes services such as medication management, symptom monitoring, professional counseling, home life, and community communication. Care-PD can become a key technology that increases the compliance of persons with PD with home care plans and improve measures to control the disease. In this article, we describe the medication management and services for PD based on the Care-PD program and its structure. The small program will improve the adverse conditions faced by persons with PD by combining the latest technology and clinical approaches. Meanwhile, we describe a verification strategy to evaluate the effectiveness of the Care-PD program as a comprehensive management strategy for PD.

## Introduction

Non-communicable diseases are a major public health challenge currently facing the world, causing poor health, economic burden, loss of life, a decline in quality of life, and poor social development in both developed and developing countries (1). Globally, 30 million people with non-communicable diseases suffer disability and death due to neurodegeneration (2). Neurodegenerative diseases lack effective treatment methods, and slowing progression is currently the recommended treatment strategy (3). PD is one of the most common neurodegenerative disease, and its incidence is second only to Alzheimer's disease (4).

The number of persons with PD in China has risen sharply. Excluding changes in the prevalence of PD, the aging population is a major concern (5). According to statistics, people over the age of 60 account for about 15% of the country's total population and this had already exceeded 200 million by 2013. By 2050, the elderly population is expected to reach one-third of the total population (6). Zhang et al. (7) evaluated 29,454 residents aged ≥55 years in 79 rural and 58 urban communities in Beijing, Xi'an, and Shanghai, and diagnosed 277 persons with PD. The incidence of PD in people >65 years old is 1.7%. Based on this, it is estimated that there are about 2.21 million persons with PD in the country, and the economic burden caused is about 17 billion RMB. As a common senile disease, the number of persons with PD will continue to grow in the foreseeable future and remain at a high level for a long time.

PD is generally treated with drugs, however, if the severity of the disease is high caregivers might be needed (8). An active home care plan is an effective strategy to cope with PD, helping persons with PD manage their condition, improve their quality of life, and live independently (9). Compared with other chronic diseases, PD management has more particularities. For example, with the popularity of glucose meters or blood pressure monitors and other monitoring instruments, patients with diabetes or high blood pressure can monitor their condition changes daily at home, which makes it easy to spot deterioration of the condition and enact timely treatment (10, 11). However, PD progresses slowly, and it is difficult for patients to accurately perceive condition changes, which delays the best time for medical treatment. In addition, PD severely reduces cognitive and memory abilities, which often causes difficulties such as taking irregular or wrong medication. As is known to all, bradykinesia is the main feature of PD, which may lead to poor compliance with the daily rehabilitation measures recommended by their doctors (12). The management of PD in a family environment also creates challenges. Therefore, medication management and rehabilitation training guidance in a family setting should be prioritized in PD treatment.

With the rapid development of information technology, the integration of medical services and network platforms has become popular. Telemedicine has gradually become an important part of home self-management for persons with chronic diseases (13), which may be the most promising method for persons with chronic diseases to implement home care plans. The popularization of smartphones among different ages has become an important prerequisite for people to actively participate in telemedicine (14). It is possible to assess and manage PD based on smartphone apps (15, 16). With appropriate training and strategies, technology can perform early screening, gait training, and management of persons with PD (17). Mobile applications have the potential to improve diagnostic efficiency and accuracy, which may be more standardized than doctors' traditional assessment methods (18).

In many countries, health care services are mainly provided in a hospital setting, which may cause transportation and time inconveniences to persons with PD (19, 20). ParkinsonNet in the Netherlands extends the professional management of PD from the hospital to the community and even to the patient's family (21). This model can provide patients with better care and save on medical care costs. It is a revolutionary management method and is attracting increasing attention. Another practical solution, a home-centered community-centered integrated care system (iCARE-PD), utilizes the potential of existing care resources and has a direct impact on clinical practice (22). iCARE-PD increases the ability to care for persons with PD by optimizing their cost, especially in rural areas or low-income countries with potential benefits (22).

A few smartphone applications have been developed to manage home-based medication and rehabilitation training for PD in China. An expert team composed of occupational therapists, graphic designers, and information technology experts has developed a mobile application called Care-PD, which has been tested and explored in reality (23). Based on previous experience, we have updated this application. This article will introduce the Care-PD 2.0 version (hereinafter abbreviated to Care-PD).

The Care-PD program is funded by the National Key R&D Program of China (No. 2018YFC1314700) to build a medicine management and service platform for PD. The Care-PD program is China's first professional medication management and rehabilitation training guidance platform for PD, which will support persons with PD to conduct self-medication, health monitoring, and rehabilitation training guidance in a family environment. To a certain extent, the application of the Care-PD program makes up for the blind use of drugs and lack of caregivers in persons with PD. Care-PD can encourage persons with PD to actively participate in and implement their medication plan and physical activity schedule recommended by their doctors, avoiding unhealthy habits. All in all, the Care-PD program may be a key measure for persons with PD to implement long-term medication management and rehabilitation training.

## The Needs of Persons With PD in China

China has the largest number of persons with PD in the world, accounting for half of the total number of patients with the disease (24). Experienced neurologists are mainly located in the top three hospitals in large cities (25), and the existing medical resources fail to meet the needs of the huge PD population. People with PD cannot receive adequate professional care from neurologists, especially in low-income and rural areas (26). This inequality due to the allocation of medical resources and regional differences is difficult to resolve quickly (27). After being diagnosed in a tertiary hospital, persons with PD are advised to return to their family or community to manage the condition according to the recommended medication plan and rehabilitation training plan. However, the doctors of the primary medical service institutions have insufficient experience in the treatment of PD, and there seems to be no treatment method other than prescribing drugs. In sum, persons with PD cannot achieve smooth referral between different medical institutions.

Individuals with PD often show different symptoms that occur at different times, and it is difficult for patients to take drugs in a standardized manner. PD treatment has complicated drug usage and poor compliance, which is very adverse for disease control (28). Although neurologists emphasized the timing and dosage of medication to persons with PD, medication errors often occur (29). PD severely reduces the memory and cognitive abilities of a person, which increases the risk of overdose, underdose, and forgetting to take the drug (30). The adverse effects of anti-Parkinson drugs should be recorded in detail, otherwise, the original medication plan cannot be adjusted in time. Besides, persons with PD cannot accurately judge the deterioration of their condition and often delay treatment (31). Due to their complicated medical history and medication experience, there are barriers to communication between outpatient doctors and persons with PD (32). For example, doctors often ask patients whether their current medications are effective. However, some patients do not know what the effective standard is, and some patients cannot remember the details of medication (33).

Exercise training has been proven to be beneficial to the motor symptoms and balance ability of people with PD (34). However, rehabilitation exercises require patients to travel between the hospital and their homes, which may cause transportation or time inconvenience to patients and caregivers. Also, persons with PD have the characteristics of being less active, so it is difficult to complete the nursing plan in a home setting, including the advice of drugs, nutrition, and sports activities. PD not only reduces the quality of life but also creates a huge psychological pressure and physical burden on their caregivers (35). The management model in a family environment aims to realize self-management of illness and active participation in rehabilitation training, thereby strengthening the independence of persons with PD and reducing the psychological pressure of their caregivers (36).

## Materials and Equipment

### Care-PD Program

The Care-PD program is China's first service platform for PD medication management and rehabilitation training. It is committed to providing individualized medication management, symptom monitoring, professional counseling, and home life and community communication services for persons with PD. At the same time, it provides doctors with auxiliary tools to manage persons with PD to improve the diagnosis and treatment experience.

### Compared With Other Chronic Disease Drug Management Platforms in China, the Care-PD Platform Has the Following Advantages

1. Provide patients with a path to self-manage their condition and encourage patients to actively describe their daily medication.
2. The main interface has detailed partitions, guiding patients to record all aspects of daily medication.
3. Logical and clear medication record helps doctors to provide an effective diagnosis and treatment.
4. Provide rehabilitation training guidance to realize convenient services in a family setting.
5. Provide self-evaluation video guidance and self-evaluation questionnaires, so that patients and healthcare professionals can fully understand their condition.

PD is a very special clinical condition, and it is often necessary to make a clear diagnosis based on the efficacy of drugs. Therefore, the treatment itself is part of the diagnosis (37). However, doctors and patients have different understandings of the evaluation of drug efficacy. The management logic of the Care-PD program provides patients and doctors with a “translation” tool, which converts the patient's continuous record into a form that is more acceptable to the doctor. And this converts the doctor's prescription in a way that the patient can understand and execute, reducing communication barriers during the diagnosis process. Overdose, insufficient medication, and forgetting to take medication are common medication problems in persons with PD. The medication management of the Care-PD program can effectively improve the medication compliance of persons with PD.

The deterioration of the original medication plan for PD indicates a worsening condition. However, the progress of the disease is easily overlooked when the symptoms are mild. Thus, persons with PD often delay the best time to see a doctor. The medication management records of Care-PD can directly reflect the efficacy of the drugs. So, doctors can find abnormalities on time and take measures to increase the benefits of persons with PD *via* Care-PD's doctor side.

**Figure 1:**
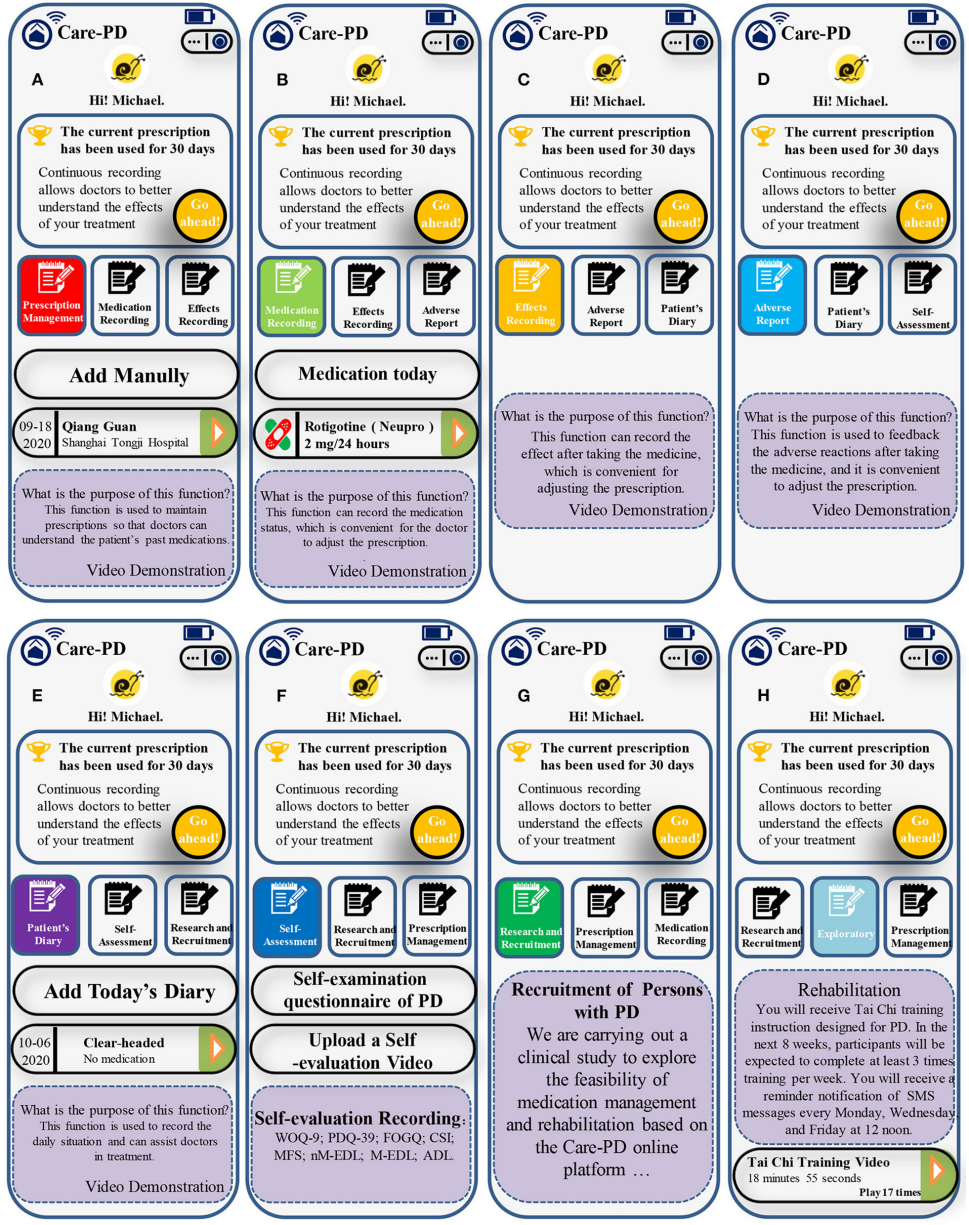
The main interface of the Care-PD program: **(A)** Prescription Management; **(B)** Medication Recording; **(C)** Effect Recording; **(D)** Adverse Report; **(E)** Patient's Diary; **(F)** Self-Assessment; **(G)** Research and Recruitment; **(H)** Exploratory.

Persons with PD record their “progress notes” continuously *via* the Care-PD program to provide a reference for future diagnosis and treatment. Recording disease conditions online is not restricted by regions and hospitals. In addition, online training based on the Care-PD program provides doctors with learning and communication opportunities. Doctors registered in the Care-PD program advocate referral cooperation, and the primary doctors' questions will be answered by high-level doctors in the team. The main interface of the Care-PD program is shown in Figure 1. The patient side of the Care-PD program includes seven basic sessions: prescription management, medication records, effect recording, adverse report, patient's diary, self-assessment, and research and recruitment.

### Prescription Management

This session helps persons with PD record every prescription adjustment during the treatment process, so that they can query prescription information. The watchman (backstage administrator) is granted permission to monitor the information filled in by persons with PD *via* the Care-PD program. Persons with PD need to choose the appropriate hospital and doctor for authorization. The doctor here is the one who issued the current prescription (the patient fills in by himself). The watchman and the prescribing doctor may be the same person or different.

1. The filled-out prescription will be displayed in the corresponding generated medication record. The medication time and medication details should be strictly filled according to the doctor's prescription.
2. The system will default the last entered prescription as the current executed prescription. If the clinician has not adjusted the treatment plan, there is no need to enter it again.
3. If the doctor changes the medication, and the medicine used was similar to the previous drug, it can be modified based on the original prescription, and a new drug will be generated after the modification is saved.
4. The newly developed prescription system will default the day after the prescription modification date as the new prescription's start date.

### Medication Recording

This session shows the Parkinson's medicines currently prescribed to be taken throughout the day and the specific medication status.

1. If you take medication on time as recommended by the prescription, you only need to select the treatment, record the time of taking the medication, and save it.
2. If you add drugs by yourself, then it should be entered manually. Please note that the added drugs are only for the off-prescription treatment of PD.
3. If drugs are added manually, patients should make sure time, type, and dosage of the drug are entered correctly.
4. Regardless of prescribed drugs or self-adding drugs, recording should be done within 24 h after taking the pills. After 24 h, the system will default to no medication on that day.

### Effect Recording

This session is used to record the actual curative effect after taking medicine and adjusting the prescription.

1. Please evaluate the efficacy of each medicine prescribed by the doctor according to your feelings.
2. If you feel that the effect is not satisfactory, please select the reason for the dissatisfaction. You can choose multiple choices or give a brief description. This is of great importance for the doctor so as to further adjust the treatment plan.
3. Efficacy evaluation can only be carried out for the medicines that are confirmed in the “recorded medication.”
4. The curative effect record needs to be entered within 24 h after taking medicine.

### Adverse Report

This session is used to give feedback on any adverse drug reaction after taking medication and to adjust the prescription.

1. If an adverse reaction occurs after taking the medicine, please choose the treatment that may cause an adverse reaction according to your real feelings. You can check the common manifestations, you can select more than one, or you can add an explanation in the text box below.
2. Please note that adverse reactions are mainly for the side effects of drugs (such as dry mouth, abdominal pain, diarrhea, and dizziness), excluding unsatisfactory curative effects, “on-off” phenomena, and abnormalities.

### Patient's Diary

This session can record the hourly condition changes throughout the day, which is a reference for doctors to adjust treatment plans.

1. Mainly for patients with more complicated conditions, the doctor will decide whether you need to register this item according to your situation, and the time of recording.
2. When recording, please follow the prompts to select the time point, awakening state, medication status, medication effect, motor symptoms, adverse reactions, and other items.

### Self-Assessment

This session provided participants with self-assessment questionnaires to assess their condition.

1. PD self-examination questionnaire is used to record the possible states of daily life and assess the risk of PD.
2. Persons with PD will complete the other eight self-rating scales, including Wearing off questionnaire-9 (WOQ-9) (38), The Parkinson's Disease Questionnaire-39 (PDQ-39) (39), Freezing of Gait Questionnaire (FOGQ) (40), Caregiver Strain Index (CSI) (41), Morse Fall Scale (MFS) (42), non-motor-experience daily living (nM-EDL) (43), motor-experience daily living (M-EDL) (43), and Activity of Daily Living Scale (ADL) (44).
3. Persons with PD will complete the assessment concerning the standardized self-evaluation guidance video and upload the self-assessment video immediately. Doctors can obtain the patient's self-assessment data and video materials through the doctor's terminal.

### Research and Recruitment

This session is used to implement a randomized controlled study to test the effectiveness of the Care-PD program.

1. After reading the recruitment plan in this section, the patient can click the “I want to join” button to conduct a preliminary assessment of ADL.
2. Participants with ADL <55 points will not be able to participate in the study.
3. Participants with ADL >55 points need to click the “I want to join” button to re-enter again, enter the detailed information of their condition, upload their diagnosis certificate, sign the informed consent form online, submit for review, and wait for the health-care professionals to evaluate whether the patient is suitable for research.
4. Participants who pass the review will be notified by text message or interface prompts, and an extra “Exploratory” module will be presented on the patient side of the Care-PD program. This part provides rehabilitation training videos for persons with PD. Persons with PD will receive reminders of rehabilitation training at least 3 times a week. The initial application of the Care-PD program is 8 weeks. Researchers monitor the participants' rehabilitation training based on the video playback time provided by the cloud.

## Realize Adaptability and Personalization Through the Care-PD Program

Generally speaking, people with PD can get help from doctors and take measures to control their condition. However, patients may ignore subtle changes in their condition, or delay the best time to see a doctor. The Care-PD program focuses on self-recording of medications for persons with PD, rational medication management, and support for better rehabilitation training guidance. Persons with PD will adapt to the medication management and rehabilitation training mode in the home environment, relying on different sessions of the Care-PD program. The Care-PD program's doctor terminal can supervise the condition changes of persons with PD, which is conducive to timely detection of adverse conditions. Moreover, the “Exploratory” module based on the Care-PD program provides different rehabilitation training modes to support patients in their active participation. The Care-PD program, as a professional management platform for PD, interactively contributes to patient self-management, health-care professional supervision, and active participation in rehabilitation training, as well as strengthening the comprehensive care of persons with PD in the family environment.

## Care-PD Program Design

### Structure

**Figure 2:**
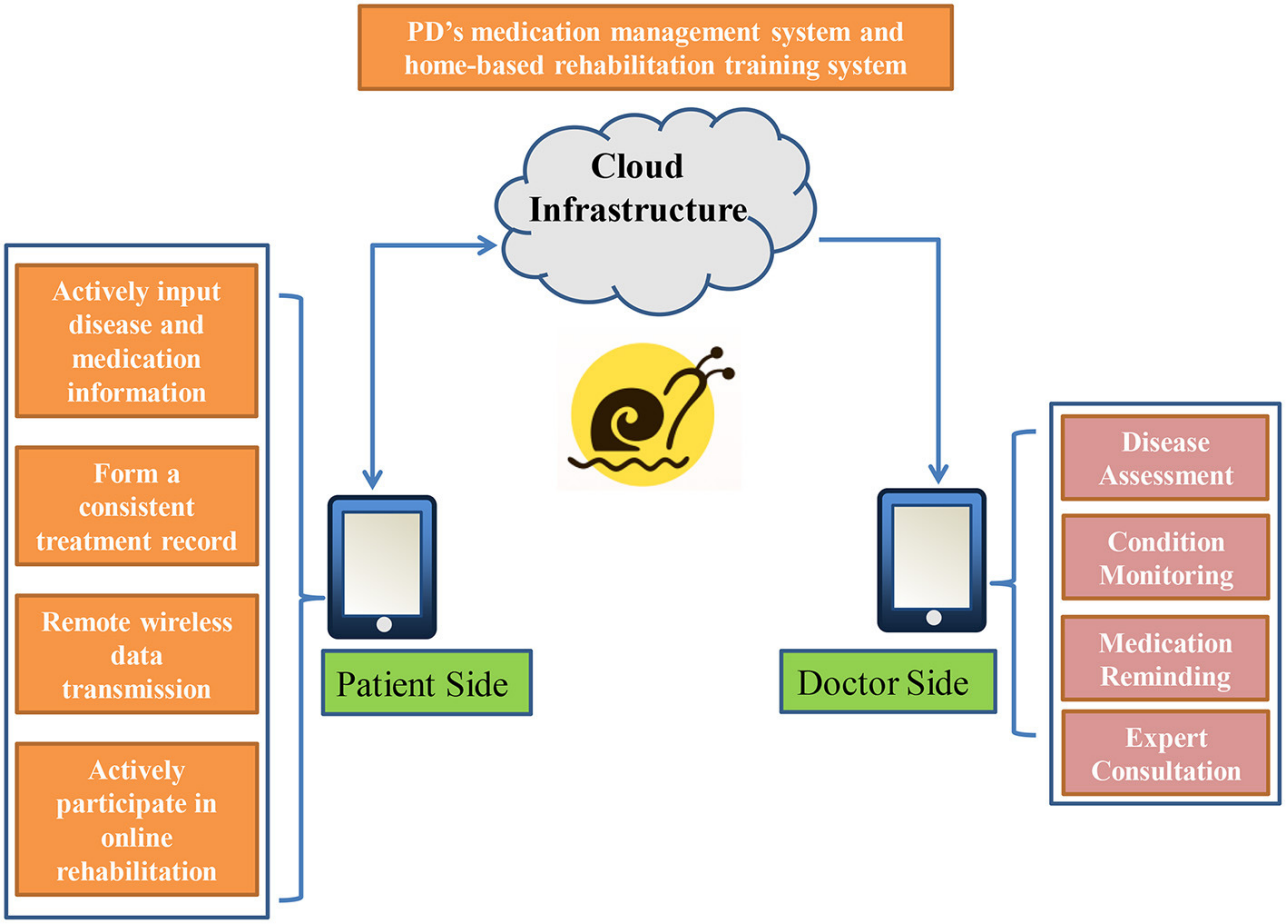
Overview of the Care-PD architecture.

The Care-PD program integrates multiple platforms and technical systems such as self-administration management, disease telemonitoring, health cloud platform, big data analysis of disease change models, and exercise program models. The system structure is depicted in Figure 2. By integrating terminal patients' condition information, the cloud platform database, and the medical care terminal applet application, doctors can make remote risk prediction for patients and provide management decision support. The Care-PD program provides self-medication management and rehabilitation training for persons with PD simultaneously.

### Workflow

**Figure 3:**
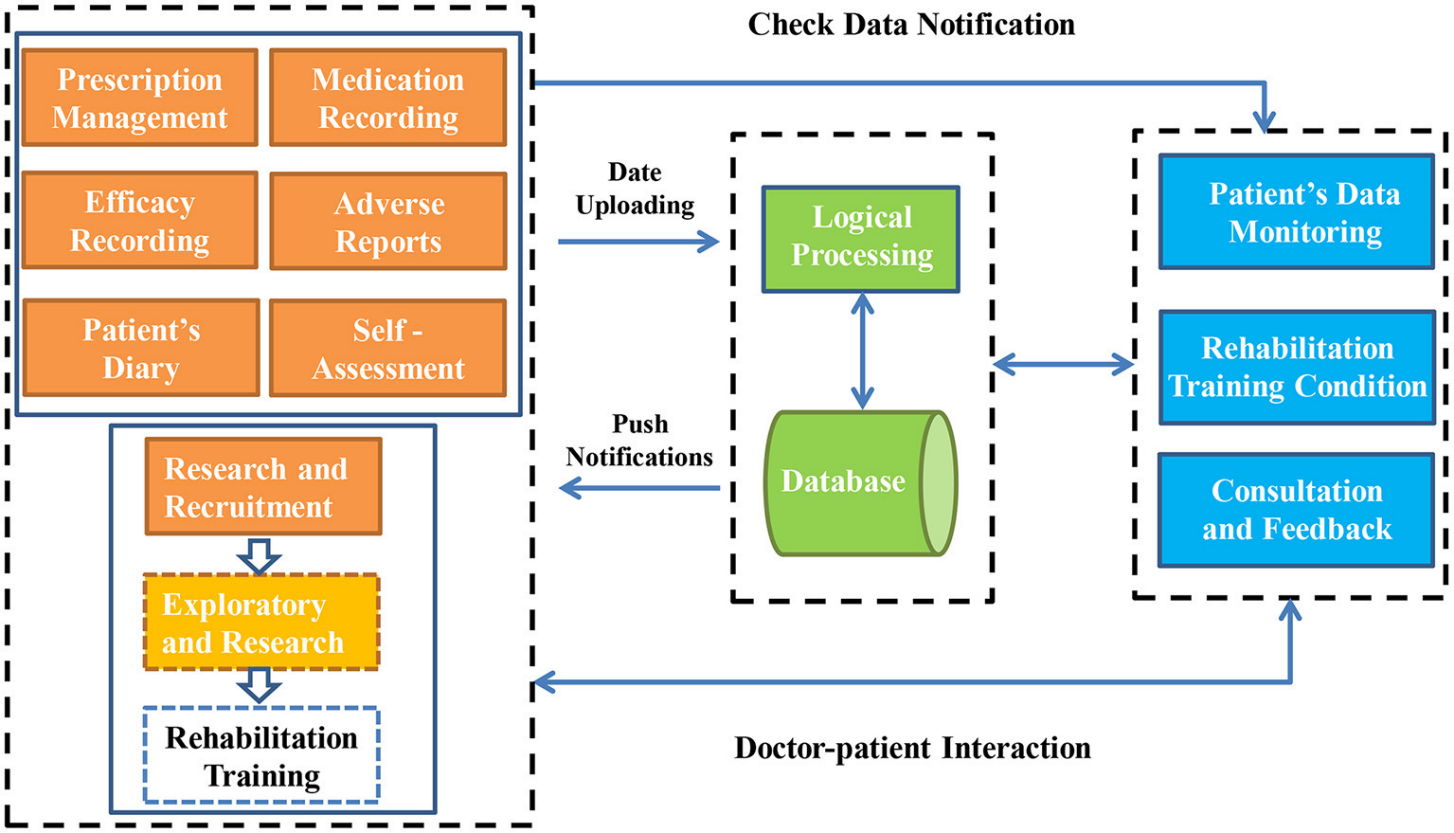
Work flow of the Care-PD program.

Persons with PD use the Care-PD program to input disease information and upload the actual disease information to the cloud server for storage. The medication reminder of the Care-PD program will offer regular prompts according to the medication plan set by the patient. Doctors can understand the actual conditions of the person with PD and evaluate the patient's condition. After being reviewed and approved by the background administrator (a qualified neurologist), those who meet the criteria for inclusion will pass the review to obtain rehabilitation training, education, and other services pushed by the mini program. The doctor's side is to monitor the medication plan and abnormal conditions of persons with PD in real-time, remind them to self-administer medication, perform rehabilitation training, evaluate the disease on time, and respond to health consultations. The system workflow is shown in Figure 3.

### Cloud Data Interaction Management and Client Implementation

**Figure 4:**
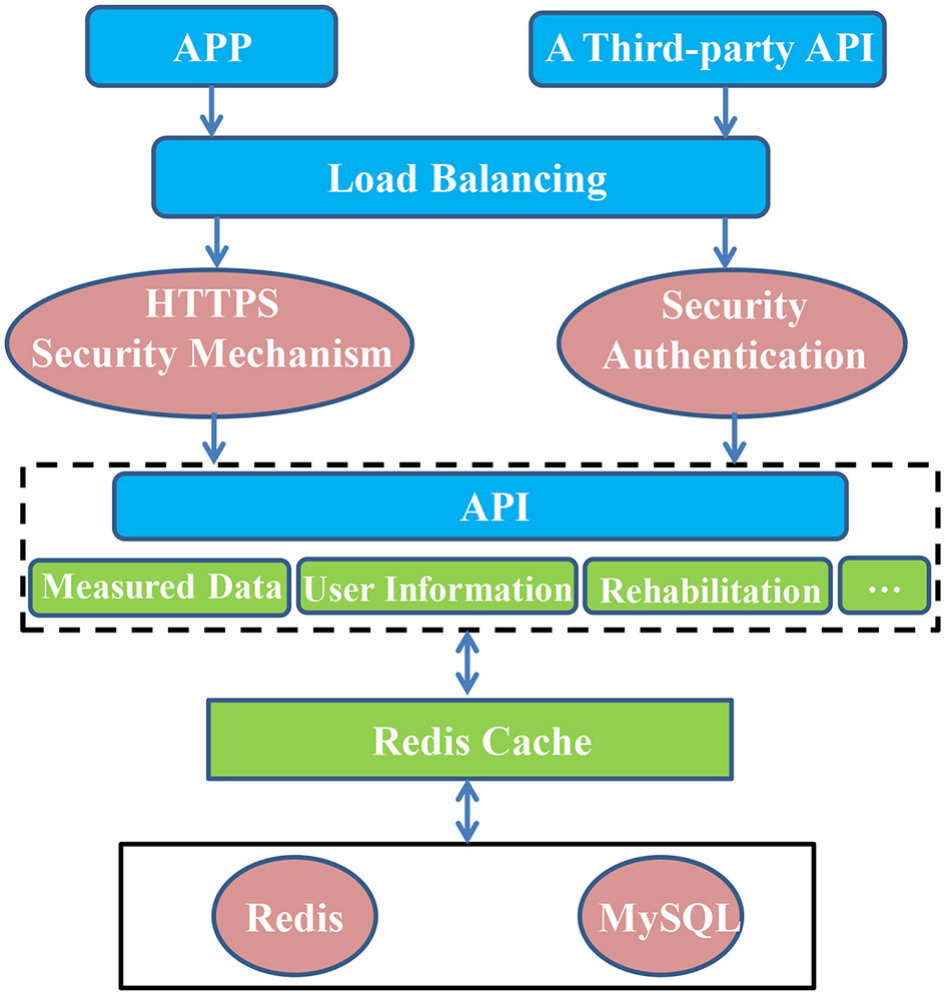
The Cloud Architecture.

The Care-PD program based on mobile medical technology is composed of client and cloud data. Data reception and management and disease risk prediction are carried out in the cloud. The Web server established based on Tomcat responds to client requests promptly and realizes the automatic reception and storage of data uploaded by users. It provides a data access interface to the user, and at the same time provides an application program interface (API) for connecting with third-party data. An exercise intervention system is established in the cloud to provide patients with rehabilitation training and push it to the mobile phone in the form of video. The server is designed with an SSH (Spring + Struts + Hibernate) framework to provide the client with user information, monitoring data interaction interface, rehabilitation plan, and education information push interface. When the client uploads data, it initiates a POST request to the server. After the HyperText Transfer Protocol Secure (HTTPS) authentication, the server responds to the request and establishes a connection with the client, parses the data, and stores it in the database. The database adopts Alibaba Cloud RDS to realize automatic database backup and disaster recovery. Using Redis as a distributed cache and a MySQL-based relational database can ensure the normal use of users in multi-user high-concurrency scenarios. The client initiates a GET request to the server when obtaining data, and the server responds to the request and returns the data to the client. Cloud architecture is depicted in Figure 4.

## Care-PD Verification

To verify the Care-PD program, it needs to determine whether the developed platform can achieve the expected purpose of medication management and active participation in rehabilitation training for persons with PD, and whether it can show that it is safe and practical. Similarly, verification must provide information on the feasibility of conducting clinical trials to prove the effectiveness of the platform in achieving the goals of self-medication management, doctor-patient communication, rehabilitation training, quality of life, and well-being for persons with PD.

First, the testing phase is carried out by the developers in the technical laboratory. The different functional components and services that build the entire platform will be integrated in the laboratory. Based on the feedback from these tests, the platform will be improved, including page design, font size, and presentation form. Whether each part of the conversation is easy to understand, whether the information can be submitted smoothly, whether the doctor's monitoring is smooth, and whether communication can be achieved also need to be checked. The purpose of the pre-evaluation is to verify whether the initial trial version of the Care-PD program meets clinical requirements, or whether its effectiveness meets individual needs. The Care-PD project conducts technical integration and verification in three different locations (Beijing, Shanghai, Shenzhen; China), and the project will release the second version of the Care-PD platform service.

Second, the clinical evaluation phase will initially involve a small number of persons with PD. Initially, a member of our research team will guide them to familiarize themselves with the care-PD program. Then, persons with PD are required to use the Care-PD program alone at home. This process is kept under the supervision of doctors and feedback can be provided by patients at any time. To evaluate the compliance of Care-PD to support the medication management and rehabilitation plan of persons with PD, the evaluation will be conducted based on clinical practice and overall experience.

Finally, a pilot study will evaluate the effectiveness of PD's medication management and rehabilitation guidance using the Care-PD program in a family environment. In this pilot study, persons with PD will be recruited based on their clinical manifestations and participate in personalized medication management and rehabilitation training programs in a family environment. This study will be set up as a randomized controlled trial in a family environment. The overall purpose of the pilot study is to test the Care-PD program to find out whether it is useful for medication management and patients to actively perform rehabilitation training activities in a family environment.

In summary, the first step is verification from a technical point of view, without patient involvement. This stage is mainly to test the correct function, infrastructure, and security of the Care-PD program. On this basis, the trial phase with a small number of patients will begin. The purpose of this stage is to evaluate the patient's usability and preference, and the system's ability to adapt to the patient's characteristics and needs in a family environment. Third, the testing process of the Care-PD program will end with the pilot testing phase, which is also (from a clinical perspective) the most important clinical verification phase that will be introduced.

The specific goal of the pilot study is to test the overall effect of medication management and rehabilitation training based on the Care-PD platform in a home environment, specifically in the form of a self-rating scale, to improve PD persons' quality of life and disease status.

This pilot study is double-blind, parallel, and RCT. Participants will be randomly divided into a control group (CG), a medication management group (MMG), and a comprehensive management group (CMG). Assessment will be carried out by persons with PD themselves at baseline and 9 weeks (end of intervention) based on the self-assessment questionnaires *via* the Care-PD platform. Recruitment is conducted online through Care-PD's WeChat public number. Persons with PD who are interested in this study can enter basic information and condition information through the “Research and Recruitment” module of the Care-PD platform and submit them for review. The platform administrator will evaluate whether the subject has passed the review based on the inclusion criteria. Randomization will be achieved through an intelligent random grouping system based on the Care-PD platform. The system presets three groups with 60 randomized people. Each participant who passes the audit will be automatically assigned to one of the three groups by the system. Only the platform manager can know how the participants are allocated. The researcher responsible for the follow-up reviews will be blinded to the distribution at any time during the data collection period. Moreover, the group assignment will be blinded to the data analysts. All recruitment procedures, including filling out questionnaires, submitting diagnosis certificates, and signing informed consent, are completed online based on the Care-PD program. Users need to have sufficient cognitive, physical, and mental health to use the system.

### Inclusion Criteria

1. Age: 40–75 years old;
2. Primary PD following the standard suggested by the Clinical Diagnostic Criteria for Parkinson's Disease in China (2016);
3. In a stable condition currently with an essential dosage of anti-PD drugs;
4. Symptoms have been relatively stable in the past month.

### Exclusion Criteria

1. Diagnosed as atypical PD syndrome;
2. Unable to stand up for 45 min due to various reasons, such as bone and joint disease, cardiopulmonary disease, or excessive weakness or fatigue;
3. The simple fall prediction questionnaire indicates that there is a high risk of falling in the next 6 months;
4. ADL <55 scores;
5. Severe cognitive or mental disorders;
6. Those who don't know how to use smartphones and whose family members cannot assist;
7. Those whose doctor in charge or investigator believes that they have other conditions that are not suitable for participating in this study.

### Intervention

Control group (CG): Participants assigned to the control group will stay on the home page of the Care-PD application until the end of the 8-week usage period. Therefore, they do not have the right to use any application functions that can help them with medication management and rehabilitation guidance, so they will be encouraged to continue their daily activities.

Medication management group (MMG): Subjects participating in the medication management group will get the function of using the application for daily medication management, but the “Exploratory” part is prohibited. Therefore, participants cannot obtain functional rights to participate in rehabilitation guidance.

Comprehensive management group (CMG): Subjects participating in the comprehensive management group are authorized to use the “Exploratory” part of the rehabilitation training function in addition to using the application for drug management. The “Exploratory” part is a set of simple Tai Chi training lessons designed for persons with PD. The training program includes 60-min exercise classes, three times a week, for 8 weeks. Each session includes 40 min of main training, 10 min of warm-up, and 10 min of a finishing exercise. For more information, a simple Tai Chi training lesson is presented in Supplementary File 1.

### Outcome Measurement

Eligible persons with PD will use the Care-PD program within 8 weeks. Each participant is required to complete the following self-assessment form at baseline and 8 weeks later. This process has been introduced in the “Exploratory” module.

1. WOQ-9 (38) is used to assess whether there is “wearing-off” during the period of taking anti-Parkinson's drugs. There are nine questions in the WOQ-9 questionnaire, with a positive prediction rate of 86%. There are two options for each symptom, and patients must have both “symptomatic” and “the symptom can be improved after the next medication” before it is counted as 1 point. If there are symptoms alone that cannot be improved after the next medication, no points are scored.
2. PDQ-39 (39) is specifically designed for PD, and includes problems encountered in life such as interpersonal relationships, social conditions, and communication. It is used to evaluate the quality of daily life of persons with PD in the past month. The higher the score, the more serious the condition.
3. FOGQ (40) is used to assess whether there is freezing of the gait of persons with PD. This questionnaire contains six items, all related to gait and walking. The higher the score, the more severe the freezing of gait.
4. CSI (41) includes patients' characteristics, the caregiver's subjective feelings about the role of caregiver, and the caregiver's psychological status. There are 13 items in total, including subjective and objective factors. The stress of the caregiver is measured in physical, mental, work, economic, and social aspects. This questionnaire adopts a yes/no answer (1 point for yes, 0 point for no). The cumulative score is ≥7 points, indicating that there is care pressure. The scale can be used for caregivers of any age.
5. MFS (42) can simply predict the risk of falling in persons with PD in the next 6 months.
6. nM-EDL (43) will be assessed by the clinically commonly used UPDRS-I scale, which has seven items; the score ranges from 0 to 28, the higher value indicates that the non-motor symptoms in daily life are more severe.
7. M-EDL (43) will be assessed by the UPDRS-II scale commonly used in clinical practice. The scale has 13 items; the score ranges from 0 to 52 with a higher value indicating that the motor symptoms in daily life are more serious.
8. The ADL (44) has 14 items, including the Physical Living Self-care Scale (six items) and the Instrumental Activity of Daily Living Scale (eight items). An evaluation result of 16 or lower could be completely normal, while more than 16 points would demonstrate different degrees of functional decline; the highest is 64 points.

### Statistical Analysis

Statistical analyses will be conducted using SPSS, version 23.0. One-way analysis of variance (continuous variables) will be used to test for differences between groups of baseline variables and the changes after the 8-week intervention. *Post-hoc* analyses using the LSD test will be applied only if the omnibus *F*-test statistics indicated that the null hypothesis should be rejected. Paired *t*-tests will be used to examine changes within the groups from baseline to 8 weeks.

### Expected Results

The overall goal of applying the Care-PD program is to improve medication management in the family environment and support rehabilitation training. The Care-PD program will be a persuasive and compelling technology designed to help persons with PD strengthen medication management and improve their rehabilitation training. Therefore, persons with PD can rely on the Care-PD program to self-manage their condition and obtain more effective disease delay. Based on the clinical goals, the expected results are divided into improving the quality of life and disease status in the family environment; better complying with the care plan. Here, we outline and briefly discuss the research objectives that have been discussed in the pilot study objectives.

### Improve the Quality of Life and Disease Status in a Family Environment

Medication management and rehabilitation guidance based on the Care-PD program is not intended to replace neurologists, but to supplement their work to ensure the continuity of care plans in the family environment. Drug management and rehabilitation in the family environment often fail due to a lack of regulation. The Care-PD program should continue to reduce medication errors and strengthen rehabilitation training for persons with PD. These factors are related to disease control.

The overall value of a family-based care plan for persons with PD is difficult to assess objectively because it is related to personal satisfaction or happiness in life. Because of this, the quality-of-life assessment is based on pre-defined standardized tests such as ADL and PDQ-39. The exploratory research session of the Care-PD program contains eight self-evaluation questionnaires commonly used in PD, involving motor and non-motor symptoms in daily life, gait, and quality of life. The doctor can evaluate the development trend of the patient's condition based on the changes in the self-evaluation of persons with PD before and after applying for the Care-PD program. Therefore, the Care-PD program may become a necessary supplement for direct contact with PD experts.

### Better Compliance With Care Plans

It is often difficult for persons with PD to follow the healthcare professionals' advice or prescribed treatment plan. There are many forms of non-compliance with medical advice: patients may miss their medication, fail to receive the recommended treatment, fail to complete the recommended rehabilitation training, adjust their medication plan by themselves, or stop treatment prematurely. Persons with PD continue to record the medication process through the Care-PD program to understand the progress of the disease, and this process is monitored by the healthcare professional in charge. Also, the exploratory research session based on the Care-PD program supports rehabilitation training guidance for PD, which greatly strengthens the compliance of the care plan in the family.

### Preliminary Findings

The fieldwork began in January 2021, including registration and baseline self-assessment by subjects through the Care-PD program. The Care-PD program has launched online recruitment of persons with PD across the country. After completing up to 8 weeks of platform usage, daily tracking, and intervention, we have learned some lessons from this short period.

The preliminary findings demonstrate two main points. The biggest problem for patients is that they do not know how to participate in the study, although a detailed registration process and recruitment process was used to inform them. This may be closely related to the age of patients, who are unable to operate smartphones proficiently so that they face difficulties in registering and entering the Care-PD platform. Second, the complex design of a certain part of the system affects the user's overall view of the system, although the system as a whole works well.

The Care-PD program provides self-medication management for persons with PD and realizes rehabilitation training guidance through exploratory sessions. From a performance point of view, the first result shows that the information users received before using the Care-PD platform is critical. When users start to use the program, they need to have a detailed understanding of the registration and operation process, because their expectations do not match their first impressions when using it. Elderly persons with PD have difficulties in operating the Care-PD program, and they need the assistance of caregivers when possible. However, it should be noted that when the participants became familiar with using the system, the testing phase of the Care-PD program stopped, and the short-term changes in the self-assessment questionnaire were not actively reflected. The following is a brief description of the participants' improvement after using the Care-PD platform for 8 weeks.

ADL, as a basic questionnaire to understand the life activity ability of patients, was not included in the scope of the analysis. Mean (±SD) between-group differences in outcomes at 8 weeks are shown in Table 1. Compared with the control group, the CMG had significantly better performance on the measures of MEDL (*P* < 0.01) and n-MEDL (*P* < 0.05) and scored lower in the CSI test (*P* < 0.05) (with lower scores indicating improvement), but did not show significant improvement in MFS, FOGQ, or WOQ-9. The CMG is also better than MMG in MEDL and n-MEDL (*P* < 0.05 for both comparisons). From baseline to 8 weeks, the MEDL of the participants in the CMG decreased by 2.11 points (*P* < 0.01), PDQ-39 decreased by 3.74 points (*P* < 0.01) (lower scores indicate improvement), and other aspects showed no significant difference. Similar improvements were observed in the MMG: mean decreases of 2.5 points in PDQ-39 (*P* < 0.05). In the CG, no significant changes from baseline were observed.

**Table 1:** Study measures at baseline and 8 weeks and between-group differences after the intervention^*^.

**Measure**	**CMG (*N* = 19)**	**MMG (*N* = 18)**	**CG (*N* = 19)**	**Between-group difference in mean change after intervention**					
				**CMG vs. MMG (95% CI)**	***P*-value**	**CMG vs. CG (95% CI)**	***P*-value**	**MMG vs. CG (95% CI)**	***P*-value**
**MEDL**									
Baseline	6.32 ± 4.00	9.39 ± 7.06	9.26 ± 5.86						
8-week	4.21 ± 3.6	8 ± 6.42	9.53 ± 6.09	−3.789 (−7.42 to −0.16)	0.041	−5.316 (−8.89 to −1.74)	<0.01	−1.526 (−5.15 to 2.10)	NS
**n-MEDL**									
Baseline	5.16 ± 3.37	7.78 ± 5.24	7.42 ± 3.81						
8-week	4.89 ± 3.00	7.33 ± 4.45	7.74 ± 3.30	−2.439 (−4.82 to −0.05)	0.045	−2.842 (−5.20 to −0.49)	0.019	−0.404 (−2.79 to 1.98)	NS
**MFS**									
Baseline	2.11 ± 2.96	3.28 ± 4.07	2.84 ± 3.02						
8-week	2.21 ± 3.48	2.61 ± 3.40	3.21 ± 3.81	−0.401(−2.76 to 1.96)	NS	−1.00 (−3.33 to 1.33)	NS	−0.599 (−2.96 to 1.76)	NS
**CSI**									
Baseline	2.05 ± 2.66	3.72 ± 3.29	2.37 ± 3.022						
8-week	1.58 ± 1.87	3.11 ± 2.89	3.74 ± 3.21	−1.532 (−3.32 to 0.26)	NS	−2.158 (−3.92 to 0.39)	0.018	−0.626 (−2.42 to 1.16)	NS
**FOGQ**									
Baseline	3.79 ± 3.38	5.83 ± 4.63	4.79 ± 4.37						
8-week	3.32 ± 3.02	4.67 ± 3.85	5.32 ± 4.39	−1.351 (−3.85 to 1.15)	NS	−2.00 (−4.47 to 0.47)	NS	−0.649 (−3.15 to 1.85)	NS
**PDQ-39**									
Baseline	25.79 ± 15.58	29.39 ± 19.43	30.37 ± 17.13						
8-week	22.05 ± 16.47	26.89 ± 17.16	30.05 ± 13.63	−4.836 (−15.26 to 5.59)	NS	−8.00 (−18.28 to 2.28)	NS	−3.164 (−13.59 to 7.26)	NS
**WOQ-9**									
Baseline	3.84 ± 2.32	4.39 ± 3.18	4.42 ± 2.27						
8-week	3.37 ± 2.57	3.56 ± 2.94	4.58 ± 2.65	−0.187 (−1.98 to 1.16)	NS	−1.211 (−2.98 to 0.56)	NS	−1.023 (−2.82 to 0.77)	NS

* *Plus-minus values are means ± SD. Significance will be set at P < 0.05. NS denotes not significant*.

## Discussion and Conclusion

In the context of coronavirus disease 2019, the development of telemedicine in a family environment has brought possibilities for medication management and rehabilitation guidance for persons with PD (45). The Care-PD program implements personalized nursing strategies in the family environment in an active participating way. However, in addition to related program optimization and software participation systems, it is important to provide persons with PD with an effective platform as a way of active medication management and rehabilitation training guidance. For these reasons, we proposed the overall architecture of the Care-PD program and conducted preliminary clinical trials to verify the effectiveness of its goals in promoting the management of PD.

Self-medication management and rehabilitation training can slow the progression of PD and is also key to improving the quality of life. To maximize the effect of delaying the disease in the family environment, the medication management and rehabilitation training of persons with PD should be active. The Care-PD program needs to be operable under different tasks and environmental backgrounds to develop into a complete medication management model and rehabilitation training guidance. Therefore, appropriate strategies must be activated to improve the medication management and rehabilitation training of persons with PD. The Care-PD program will continue to provide self-administration channels, doctor supervision channels, and rehabilitation training guidance to support persons with PD to improve their physical functions and health habits. By doing so, the factors that restrict patients from returning home or managing their illness will be reduced. In the digital age we live in, technological advancement should provide a solid foundation for the development of a new chronic disease management platform. These practices can help persons with PD improve their quality of life and health.

We found that a comprehensive management program based on the Care-PD platform for 8 weeks, as compared with an MMG or a CG, was effective in improving motor and non-motor experience daily living in persons with PD. Clinically, these changes indicate increased potential for effectively performing daily life functions, such as wearing clothes, writing, transitioning from a bed to a standing position (and from standing to lying), going to the bathroom, and sleeping. However, the MMG and the CG did not show a significant difference. The short application time of the platform may not reflect the effectiveness of the pure medication management function. Participants or their caregivers are not familiar with the operation of the platform, which may be another reason that seriously reduces the management effect. In addition, the CMG showed significant differences in CSI, which indicates that comprehensive management based on the Care-PD platform can reduce the pressure on caregivers. After 8 weeks using the platform, both the CMG and the MMG showed improvements in PDQ-39, indicating that simple medication management or comprehensive management may improve the quality of life of PD to a certain extent.

However, the application of the Care-PD program has certain limitations. It requires patients to have a certain level of education or the supervision of family caregivers, moreover it also necessary to have certain daily living activities to complete the training program. Therefore, at present, the Care-PD program cannot assist all persons with PD, especially those with serious conditions. However, these problems are unsolvable for persons with PD using a mobile device. The randomized controlled study carried out is based on the remote intervention of the Care-PD program, and the scale scores before and after the intervention can only be obtained by the way of patient's self-assessment, so subjectivity in the process of patient self-assessment is inevitable. In addition, since the recruitment and randomization of this study are completely network-based, the baseline demographic information (categorical variables) of the participants was not tested, which may affect the objectivity of the results. The 8-week program usage can only be used as a preliminary test, and longer-term applications and feedback will be carried out in the future. It is expected that the steps from registration to submission for review will be more intelligent, such as using voice responses or prompt sounds to guide patients to register and add information. The Care-PD program is a new exploration tool to manage PD in the family environment, and the shortcomings will continue to be improved in future research and practice. A more user-friendly and convenient design will facilitate the wide application and promotion of the Care-PD program in persons with PD.

## Data Availability Statement

The original contributions presented in the study are included in the article/Supplementary Material, further inquiries can be directed to the corresponding authors.

## Ethics Statement

The studies involving human participants were reviewed and approved by the Ethical Review Committee of the Shanghai University of Sport (Approval Number: 102772020RT132). The patients/participants provided their written informed consent to participate in this study.

## Author Contributions

SG, YH, and RM directed the project, writing, and submission. KK participated in development and writing. LJ, HW, and RW contributed to the development, procedure, and funding for research. All authors agree to be accountable for all aspects of the work and gave final approval for this version to be published.

## Conflict of Interest

The authors declare that the research was conducted in the absence of any commercial or financial relationships that could be construed as a potential conflict of interest.
