# Supplementary material for: A Novel Management Platform Based on Personalized Home Care Pathways for Medicine Management and Rehabilitation of Persons With Parkinson's Disease—Requirements and Implementation Plan of the Care-PD Program
Source: Front Neurol. 2021 May 25;12:672208. doi: 10.3389/fneur.2021.672208 (PMC8186830; doi:10.3389/fneur.2021.672208)
Supplement: Supplementary file 1 [file Data_Sheet_1.PDF]

Introduction to simple Tai Chi program

Our team members designed the simple Tai Chi (STC) program for patients with mild to moderate Parkinson's disease in 2019. The simple Tai Chi program is easy to learn, easy to remember. It retains the essential movement elements of traditional Tai Chi, such as holding the ball, horse step, pushing palms, pressing palms, and changing the center of gravity. Between October 2019 and November 2019, the STC has been performed in five PD patients who were invited to learn and practice the STC training program for 4weeks. The STC training program has been further modified according to the comments and the patients' feedback before the STC final version. The simple Tai Chi program includes four types of movements, ten activities, designed for PD patients with balance, flexibility, strength, coordination, and other obstacles.

(I) **Preparing and Starting form**

Stand on the posture of infinity shoulder with apart.

Head, neck, and Spine perfectly aligned.

Shift weight to the right leg and step out with the left leg.

Center the body weight on both legs.

Bend arms 90 degrees by the ribcage with hands forming fists.

Breath naturally and get ready to start tai-chi.

Participants adjust their breathing, relax and prepare for training in a steady state.

(II) Up and down form

Stand on an anatomical position facing straight forward.

Transfer weight to right leg then Left foot move to the left heel up then step with toes first creating shoulder with apart.

Both arms straight and raised to shoulder level with palms facing up.

Then rotate the palms to face down as pressing down and bringing the hands down.

Raise both arms laterally above the head then bring them forward fully stretched in front.

Lower them with palms facing down, then raise them above the head and back to the lateral position still straight repeat this four times.

This action is conducive to increasing the flexibility of the participants' shoulder joints, thereby expanding the range of motion of the shoulder joints.

(III) Bend over form

Stand shoulder with apart with arms.

Elbows of both arms bend approximately 90 degrees in front of the body, hands forming fists.

Half rotate your body to the left with an open fist as you straighten the left arm.

Right arm still bend, the upper body bend over until the straightened left-hand reaches angle of the left leg .

Rotate upper body to the right with an open palm facing down of the left hand, rotating the upper body to the right slowly while still bending.

Straighten up slowly as forming the fist with the left arm .

Back to the starting position.

Repeat the same movement but in a different direction.

This action is conducive when strengthening the core muscle groups' stability, increasing the ductility of the posterior thigh muscle groups ,and overall body coordination.

(IV) Curled spine form

Stand shoulder with apart on anatomical position.

Raise both arms slowly laterally outward until they reach the back of the head.

Hold the end of the head with both hands then curl the trunk until you get the knees to hold for some seconds.

Straighten up the box slowly then leave the head.

Arms come down slowly with palms facing down back to the anatomical position again.

Repeat the movement four times.

This action helps to increase the range of motion of the shoulder and elbow joints, stretch the back of the leg, and improve the leg muscles' flexibility.

(V) Cross wrists form

Stand on anatomical position.

Open legs shoulder with apart with knees bent 90 degrees.

Arms bend by the ribcage with palms forming fists.

Bring arms together in front by chest level crossed, still with fists.

Push them in and in a downwards direction, even crossed then push them outward as you straighten the arms.

Then go back to the starting position.

This action is beneficial for increasing the flexibility of the wrist and the quadriceps strength.

(VI) Go through palms form

Standing shoulder with apart turn to the left with left foot slightly bend and right foot straight.

Straighten the left arm slowly as you turning to the left with the open palm facing outward.

Withdraw the left arm back to the ribcage as you continue to do the same movement on the right side.

Withdraw legs slowly back to the starting position.

This action helps to increase the rotation angle of the forearm, the strength of the quadriceps and the ankle's flexibility.

(VII) Standing on one leg

Stand with legs together then open shoulder with apart slowly.

While laterally raising both hands outwards.

Slightly lift the right leg as it bends 45 degrees to touch the back of the left leg.

Arms at this time are straight but turn somewhat in front of the body with palms facing inward.

Bring both legs and arms together as attaining the anatomical position.

Repeat the same movement with the left leg.

This action is beneficial to increase the strength of the quadriceps, core stability, and balance ability.

(VIII) Flying form

Move one-step leftward in a horizontal direction, tiptoe on the floor firstly.

Erect upper body to be straight; Lift the heel, lift arms outwards.

Heel drop, with both arms close to the front of the chest; Lift the knee and lift arms outwards. Knee drop. Both arms close to the front of the chest.

This action is beneficial to increase the strength and balance ability of the quadriceps.

(IX) Opening hands and closing hands

Lift hands, both palms up, to shoulder level.

Transfer the center of the body to the right; Close both hands.

Withdraw left foot and both arms.

The action in the opposite direction is the same.

This action is beneficial to increase the strength and balance ability of the quadriceps.

(X) Closing form

Lift hands, both palms up, to chest level, inhale.

Turn over palms and press down, exhale.

Transfer the center of the body to the right, withdraw the left foot.

Relax and breathe naturally.

Participants adjust their breathing, relax their bodies, and end training.
